# Microbial Community Response to Polysaccharide Amendment in Anoxic Hydrothermal Sediments of the Guaymas Basin

**DOI:** 10.3389/fmicb.2021.763971

**Published:** 2021-12-10

**Authors:** Viola Krukenberg, Nicholas J. Reichart, Rachel L. Spietz, Roland Hatzenpichler

**Affiliations:** ^1^Department of Chemistry and Biochemistry, Montana State University, Bozeman, MT, United States; ^2^Thermal Biology Institute, Montana State University, Bozeman, MT, United States; ^3^Center for Biofilm Engineering, Montana State University, Bozeman, MT, United States; ^4^Department of Microbiology and Cell Biology, Montana State University, Bozeman, MT, United States

**Keywords:** metabolic activity, heterotrophic community, microbial ecology, substrate analog probing, bioorthogonal non-canonical amino acid tagging, fluorescence-activated cell sorting

## Abstract

Organic-rich, hydrothermal sediments of the Guaymas Basin are inhabited by diverse microbial communities including many uncultured lineages with unknown metabolic potential. Here we investigated the short-term effect of polysaccharide amendment on a sediment microbial community to identify taxa involved in the initial stage of macromolecule degradation. We incubated anoxic sediment with cellulose, chitin, laminarin, and starch and analyzed the total and active microbial communities using bioorthogonal non-canonical amino acid tagging (BONCAT) combined with fluorescence-activated cell sorting (FACS) and 16S rRNA gene amplicon sequencing. Our results show a response of an initially minor but diverse population of *Clostridia* particularly after amendment with the lower molecular weight polymers starch and laminarin. Thus, *Clostridia* may readily become key contributors to the heterotrophic community in Guaymas Basin sediments when substrate availability and temperature range permit their metabolic activity and growth, which expands our appreciation of the potential diversity and niche differentiation of heterotrophs in hydrothermally influenced sediments. BONCAT-FACS, although challenging in its application to complex samples, detected metabolic responses prior to growth and thus can provide complementary insight into a microbial community’s metabolic potential and succession pattern. As a primary application of BONCAT-FACS on a diverse deep-sea sediment community, our study highlights important considerations and demonstrates inherent limitations associated with this experimental approach.

## Introduction

Hydrothermal vent fields provide areas of high biodiversity and primary productivity within a predominantly oligotrophic sea. In the Guaymas Basin, Gulf of California, active seafloor spreading and high sedimentation rates result in a unique system of hydrothermal vents within sediments with up to 4% organic matter content (Calvert, 1966; De la Lanza-Espino and Soto, 1999). Fresh organic matter derived from high phytoplankton productivity in surface waters and terrestrial runoff, is introduced into sediments while hydrothermal pyrolysis transforms and remobilizes buried organic matter, creating hydrocarbons and low molecular weight compounds that are mobilized via hydrothermal circulation (Calvert, 1966; Von Damm et al., 1985; De la Lanza-Espino and Soto, 1999). The wide range of available organic and inorganic substrates sustains a metabolically versatile anaerobic microbial community including heterotrophs and chemolithoautotrophs, and populations that typically occur only in geographically distant hydrothermal vents, cold seeps and organic-rich sediments (Teske et al., 2016, 2021). Recent metagenomic surveys detected many previously unknown and uncultured lineages and indicated that much of the metabolic diversity of the microbial community remains to be discovered (Dombrowski et al., 2017, 2018; Baker et al., 2021).

Revealing the metabolism of uncultured microbes and their function in the environment remains a challenge and frequently relies on combinatorial approaches to link taxonomic identity with metabolic activity on a single cell level. Stable isotope probing combined with fluorescence *in situ* hybridization and nanoscale secondary ion mass spectrometry or Raman micro-spectroscopy allows tracking incorporation of growth substrates into biomass of individual taxonomically identified cells (Huang et al., 2007, 2009; Dekas and Orphan, 2011; Berry et al., 2015; Musat et al., 2016). While powerful, this approach relies on isotope-labeled substrates that are not generally available for experimental testing, especially in the case of complex compound classes. Substrate analog probing is a promising but comparatively new alternative to stable isotope probing (Hatzenpichler et al., 2020). Currently, the most widely used substrate analog probing approach in microbial ecology is bioorthogonal non-canonical amino acid tagging (BONCAT) in combination with fluorescence-activated cell sorting (FACS). BONCAT-FACS can be combined with 16S rRNA gene amplicon sequencing or metagenomics to assess the anabolically active fraction of a microbial community. BONCAT uses a non-canonical amino acid, such as *L*-homopropargylglycine (HPG), that is incorporated into newly synthesized proteins by methionyl-tRNA synthetase, and that can be fluorescently detected via azide-alkyne click chemistry (Kiick et al., 2002; Hatzenpichler et al., 2014). Subsequently, FACS enables the separation of translationally active (BONCAT positive) cells for taxonomic identification via gene sequencing. BONCAT-FACS was recently applied on environmental and human microbiome samples to reveal anabolically active community members and to test responses in metabolic activity upon substrate amendments (Hatzenpichler et al., 2016; Couradeau et al., 2019; Reichart et al., 2020; Riva et al., 2020; Valentini et al., 2020; Marlow et al., 2021; Taguer et al., 2021). Here, we applied this approach to investigate the microbial community involved in anaerobic polysaccharide mineralization in Guaymas Basin sediments.

Guaymas Basin sediments contain a variety of organic macromolecules (lipids, proteins, DNA and polysaccharides) (De la Lanza-Espino and Soto, 1999; Teske et al., 2002; Zheng et al., 2021) and harbor a diverse heterotrophic community with the genetic potential to degrade a wide range of these compounds (Dombrowski et al., 2018; Baker et al., 2021; Perez Castro et al., 2021). Polysaccharides are abundant in the ocean and are commonly introduced into sediments from surface waters or terrestrial runoffs or are produced from decaying biomass. We focused on testing the potential for anaerobic microbial degradation of four polymers: cellulose, chitin, laminarin, and starch, which serve to represent marine and terrestrial polysaccharides of differing complexity that are introduced into Guaymas Basin sediments due to very high sedimentation rates and terrestrial input (Calvert, 1966; De la Lanza-Espino and Soto, 1999). Laminarin and starch are storage polysaccharides found in marine macroalgae, phytoplankton and microbial cells, while the structural carbohydrates cellulose and chitin are components of fibers and exoskeletons (Arnosti et al., 2021). The utilization of polysaccharides relies on extracellular carbohydrate-activating enzymes (CAZymes) for the initial cleavage and uptake through membrane transporters prior to intracellular degradation. Substrate-specific CAZymes such as for starch (alpha-amylase) or cellulose (glycoside hydrolase) are synthesized by specialized functional guilds while the produced oligo- and monosaccharides or intermediates of their degradation provide substrates for other metabolically diverse community members. Here, we exposed anoxic hydrothermal sediments to polysaccharides for a short time period and analyzed shifts in the overall community and active community via BONCAT-FACS combined with 16S rRNA gene amplicon sequencing providing complementary insight into primary responders and response succession.

## Materials and Methods

### Sample Collection and Material Selection

Hydrothermally heated sediments were collected with push cores in the Marker 14 hydrothermal area in the Guaymas Basin, Gulf of California, Mexico during R/V *Atlantis* research cruise AT42-05 (November 2018) with the submersible *Alvin* (Dive 4998; 2011 m; 27.0079° N, 111.4072° W). *In situ* temperature profiles were recorded using *Alvin’s* heat flow probe adjacent to the sampled sediment cores every 10 cm down to a depth of 50 cm. Two of the retrieved sediment cores (11 and 12; Supplementary Figure 1) were sectioned into three horizons: (H1) upper horizon 0–10 cm (excluding bacterial mats), 4–33°C; (H2) middle horizon 10–20 cm, 33–74°C; (H3) lower horizon, 20–30 cm, 74–110°C (Supplementary Table 1). Replicate horizons from both cores were combined into glass bottles, degassed with N_2_, and stored under N_2_ headspace at 4°C until further processing (February 2020).

As fresh organic matter is frequently introduced into surface sediments from the overlaying water column, sediments of the upper horizon were selected for mesocosm experiments testing the microbial community’s potential to utilize complex carbon polymers. Furthermore, due to the steep temperature gradient and elevated temperatures in the lower horizons, cell numbers rapidly decreased with sediment depth. Initial cell extraction tests showed that sufficient cells needed for the BONCAT-FACS approach could only be recovered from the upper sediment horizon. This highlights that selection and preliminary evaluation of the starting material is crucial in designing experiments for BONCAT-FACS.

### Mesocosm Setup

Mesocosms were prepared in an anoxic glove box under N_2_/CO_2_/H_2_ (90%/5%/5%) atmosphere. Sediment of the top horizon (H1) was diluted (1:5) with anoxic artificial seawater (Widdel and Bak, 1992). During experiment set up the slurry was homogenized by constant stirring using a magnetic plate and stir bar. To preserve material for DNA extraction, three aliquots (1 mL each) were centrifuged 10 min at 16,000 × *g*, supernatant was decanted, and pellets were frozen at −80°C until further processing. To set up mesocosms, the remaining slurry was distributed in aliquots of 30 mL into 156 mL sterile serum vials using sterile serological plastic pipettes. All vials were sealed with a butyl rubber stopper and aluminum crimp. The headspace was degassed with N_2_/CO_2_ (90%/10%) at 100 kPa for 5 min to remove H_2_ introduced during set up inside the glove box and the final headspace was set to 200 kPa. The vials were placed at 33°C, which conforms with the upper *in situ* temperature measured in the sediment horizon, for 16 h to allow the microbial community to adapt to the incubation temperature after long-term storage at 4°C. After this preincubation, the following treatments were set up, each in triplicate: (1) no substrate amendment, or amendment with (2) cellulose, (3) chitin, (4) laminarin, or (5) starch. Substrates were added from sterile anoxic stock solutions to final concentrations of 0.01% w/v. The amino acid analog *L*-homopropargylglycine (HPG; Click Chemistry Tools, Scottsdale, AZ) was added to all treatments from an anoxic stock solution to a final concentration of 100 μM. Controls were prepared without HPG and without substrate amendment. Incubation was continued at 33°C and subsamples were collected after two (T1) and five (T2) days from each mesocosm using a syringe and needle. Subsamples (1 mL each) were preserved for DNA extraction by centrifugation (10 min at 16,000 × *g*), supernatant removal, and storage at −80°C. Replicate subsamples (2.5 mL each) were preserved for cell extraction by transfer into sterile 10% Glycerol Tris/EDTA (GlyTE) solution and storage at −80°C.

### DNA and Cell Extraction

DNA was extracted from sediment pellets (see above) using the FastDNA Spin Kit for Soil (MP Biomedicals, Irvine, CA) following the manufacturer’s guidelines. Blank DNA extractions were performed as a negative control. DNA extracts were quantified using the Qubit high sensitivity assay (Invitrogen, Carlsbad, CA) and were stored at −20°C.

Cells were extracted from duplicate 2.5 mL aliquots preserved in GlyTE (see above). For this, samples were thawed at 4°C, amended with 7.5 mL of sterile 1× phosphate buffered saline (PBS; filter-sterilized (0.2 μm pore size) and UV treated for 16 h) containing Tween 20 at a final concentration of 0.02% and vortexed for 10 min at speed 8 (approx. 2000 rpm, Vortex-Genie2, Scientific Industries, Inc., Bohemia, NY, United States) to detach cells from particles. Blank extractions using 1 × PBS were processed in parallel to assess contamination introduced during the extraction process. A density gradient separation was performed by carefully adding an equal volume (10 mL) of Nycodenz solution (60%) underneath the sample using a sterile serological pipette and centrifugation at 14,000 × *g* for 45 min. Following centrifugation, avoiding any sediment particles in the lower phase, the upper phase and interphase containing the cells were transferred to a new tube prefilled with 20 mL 1 × PBS. To collect cells, the sample was centrifuged 10 min at 16,000 × *g*, supernatant was carefully removed, and the cell pellet resuspended in the remaining liquid (approx. 1 mL). Subsequently, the sample was transferred to a 1.5 mL tube and cells were concentrated by centrifugation for 10 min at 18,000 × *g*, supernatant removal, and resuspension in 50 μL of 1 × PBS. The cell suspensions from duplicate extractions were combined and immediately further processed for FACS.

### Click Reaction, Fluorescence-Activated Cell Sorting and Cell Lysis

Cell extracts were subjected to click reaction as previously described (Hatzenpichler and Orphan, 2015; Reichart et al., 2020). For this, 200 μL of click reaction solution were added to and mixed with the cell extract by pipetting up and down. The cell click reaction mixture was comprised of 5 mM aminoguanidine hydrochloride (Sigma-Aldrich, St. Louis, MO), 5 mM sodium *L*-ascorbate (Sigma-Aldrich, St. Louis, MO), 100 μM copper sulfate pentahydrate (Sigma Aldrich), 500 μM THPTA (Click Chemistry Tools, Scottsdale, AZ) and 4 μM Cy5 picolyl-azide dye (Click Chemistry Tools, Scottsdale, AZ), and was incubated for 40 min in the dark at room temperature. Subsequently, the click reaction solution was removed from the cells by three cycles of centrifugation at 18,000 × *g* for 5 min, aspiration of the supernatant by pipette, and resuspension of the cell pellet in 1 mL of 1 × PBS. To remove any remaining larger particles prior to cell sorting, a slow centrifugation was performed at 500 × *g* for 5 min, followed by passage of the supernatant through a 35 μm pore size filter cap (BD-falcon cell strainer cap, Corning, Corning, NY, United States). A subsample (50 μL) of the final cell suspension (filtrate) was transferred to a new tube and was stored at 4°C until further processing. This subsample represents the total extractable cells in our experiment. Fluorescence signal intensity and density of labeled cells was checked by epifluorescence microscopy (Leica DM4B microscope).

Cell sorting was performed using a Sony SH800S FACS (Sony Biotechnology, San Jose, CA) equipped with a 70 μm chip, set to detect Cy5 dye on the red channel (excitation 638 nm) and operated with 1 × PBS (sterilized as described above) as sheath fluid. Sorting gates were established as previously described (Reichart et al., 2020). Briefly, two gates were drawn based on forward and back scatter to exclude any larger particles and background noise; the third gate was drawn based on forward scatter and Cy5 fluorescence intensity to capture BONCAT positive cells (i.e., the fluorescent, translationally active cell fraction). Cell extracts from control incubations without HPG (no BONCAT signal) were used to delineate the gates for BONCAT positive and BONCAT negative cells. Each sample was sorted for a maximum of 250,000 fluorescent events from the BONCAT positive (active) fraction or until the volume was exhausted (Supplementary Figure 2). Sorted cells were stored at 4°C for up to 8 h until further processing.

Cells in the sorted and extractable cell fractions were lysed for DNA recovery as previously described (Reichart et al., 2020). In short, cells were centrifuged at 18,000 × *g* for 5 min followed by supernatant removal and pellet resuspension in 20 μL of nuclease-free water. Cell suspensions were transferred into 96-well plates, sealed with adhesive sterile aluminum foil and subjected to three cycles of freezing (−80°C, 10 min) and thawing (99°C, 10 min) with a short centrifugation prior to each freezing step. Lysed cell extracts were stored at −80°C until further processing.

### 16S rRNA Gene Amplification and Sequencing

Amplification of bacterial and archaeal 16S rRNA genes was performed following the Earth Microbiome protocol (Thompson et al., 2017) using revised primers 515F (5′-GTGYCAGCMGCCGCGGTAA-3′) (Parada et al., 2016) and 806R (5′-GGACTACNVGGGTWTCTAAT-3′) (Apprill et al., 2015) with DNA templates recovered from either (1) sediment samples using a DNA extraction kit or (2) cell suspensions of extractable and sorted fractions using freeze/thaw cycles (described above). Polymerase chain reaction (PCR) was performed in a final volume of either (1) 25 μL consisting of 5 μL template from extraction kits (1 ng DNA/μL), 10 μL Invitrogen Platinum Taq II 2X Master Mix, 0.5 μL (0.2 μM) of each primer, and 9 μL nuclease-free water or (2) 50 μL consisting of 20 μL template from freeze/thaw extractions, 20 μL Invitrogen Platinum Taq II 2X Master Mix, 1 μL (0.2 μM) of each primer, and 8 μL nuclease-free water. The thermocycler conditions were: 94°C for 3 min followed by 28 cycles of 94°C for 45 sec, 50°C for 60 sec, and 72°C for 90 sec, followed by a final elongation step at 72°C for 10 min. PCR on sorted and extractable cell fractions from T1 was performed with two additional cycles (for a total of 30 cycles) due to lower cell recovery. A negative control using nuclease-free water as template was included in each PCR set to determine PCR contamination. PCR products were checked for the expected length on a 1% agarose gel and subsequently were purified using AMPure XP beads (Beckman Coulter, Brea, CA, United States) following the manufacturer’s protocol with a final elution volume of 40 μL nuclease-free water. To prepare amplicons for Illumina sequencing, Illumina dual index barcode and sequencing adapters were attached in a second PCR reaction. This PCR was performed in a final volume of 25 μL containing 5 μL of purified amplicons, 12.5 μL Invitrogen Platinum Taq II 2X Master Mix, 2.5 μL (0.25 μM) of each primer (i5 and i7), and 2.5 μL nuclease-free water. The PCR conditions were as follows: 95°C for 3 min followed by 8 cycles of 95°C for 30 sec, 55°C for 30 sec, and 72°C for 30 sec, followed by a final elongation step at 72°C for 5 min. PCR products were purified using AMPure XP beads as described above and were quantified in triplicate reactions using the Quant-iT Picogreen dsDNA Assay (Invitrogen) on a Biotek Synergy H1 Hybrid microplate reader following the manufacturer’s guidelines. Purified amplicons from all samples were pooled at 10 ng DNA each, and the pooled library was quantified with the Qubit high sensitivity assay (Invitrogen, Carlsbad, CA, United States). Purification, quality assessment and sequencing of pooled libraries was performed at the Molecular Research Core Facility at Idaho State University (Pocatello, ID) using Illumina’s MiSeq chemistry for 2 × 250 bp paired end reads. Sequences are deposited at NCBI’s GenBank under BioProject PRJNA748056.

### Bioinformatics Data Analyses

Amplicon data were processed using QIIME 2 version 2020.2 (Bolyen et al., 2019). In short, primer sequences were removed from demultiplexed reads using *cutadapt* (Martin, 2011) with an error rate of 0.12. Reads were truncated to 145 bp and subsequently filtered, denoised and merged in DADA2 with default settings (Callahan et al., 2016). The resulting 16S rRNA gene amplicon sequence variants (ASVs) were taxonomically classified with the *sklearn* method and the SILVA 132 database (Quast et al., 2013). Contaminating ASVs were removed using the R package *decontam* with the prevalence model at a threshold of 0.6 followed by manual inspection and further curation considering multiple controls included in the dataset (i.e., PCR negative controls, DNA extraction blanks, sheath fluid controls) (Supplementary Figure 3). Samples with less than 10,000 reads were excluded and ASVs with less than 0.001% relative sequence abundance across all samples were removed prior to further analysis. Diversity metrics were calculated on the level of ASVs in the R package *Phyloseq* (McMurdie and Holmes, 2013). ANOVA-like differential gene expression analysis was conducted in the R package *Aldex2* (Fernandes et al., 2013, 2014) between no amendment control and each substrate treatment on the level of ASVs. Adjusted *p*-values <0.05 were considered for calling differential abundance of ASVs (Supplementary Table 2).

## Results and Discussion

### Sampling Site Description and Experimental Strategy

Sediment for mesocosm experiments was recovered from the Marker 14 site in the Guaymas Basin, an area with strong hydrothermal influence (Supplementary Figure 1). The sediments were covered with orange *Beggiatoa* mats, and were characterized by steep temperature gradients reaching 115°C at 35 cm depth (Supplementary Table 1). Further, sulfide and ammonia concentrations ranged between 2–4 mM and 2–6 mM, respectively, and sulfate concentrations decreased with depth (Ramírez et al., 2021). Previously recorded *in situ* microprofiles from the same area showed rapid oxygen depletion in the upper two sediment millimeters, with occasional deeper oxygen traces due to hydrothermal circulation (Teske et al., 2016).

In mesocosm experiments we exposed sediments of the upper horizon to polysaccharides of differing complexity for a limited time period (2 and 5 days) to identify populations involved in the initial polysaccharide activation and utilization under anoxic conditions. While substrate-specific enrichments select for specialized communities over time, they may not reveal the community members first responding to a substrate. As metabolic activity precedes growth, tracking the active community members via BONCAT-FACS after short-term exposure to a substrate can provide complementary insight into the primary degraders and degradation succession. To identify substrate-specific responses we compared the active and total communities in substrate amended mesocosms to controls without amendments. Notably, the studied sediments are naturally rich in organic and inorganic compounds that support microbial metabolism. We analyzed the microbial community composition in three fractions obtained by (1) DNA extraction, (2) cell extraction, and (3) cell sorting, which are considered representative of (1) the total DNA extractable community, (2) the total cell extractable community, and (3) the translationally active, cell extractable community. As the DNA extractable (1) vs cell extractable (2, 3) fractions result from different extraction processes an unbiased representation of the *in situ* community cannot be provided. Furthermore, assessment of the active fraction is affected by limitations associated with BONCAT and FACS. Finally, primer-based PCR amplification and sequencing introduce additional biases; thus, all relative sequence abundance data is limited in its representation of community composition.

### Diversity and Composition of the Total and Active Communities

**FIGURE 1:**
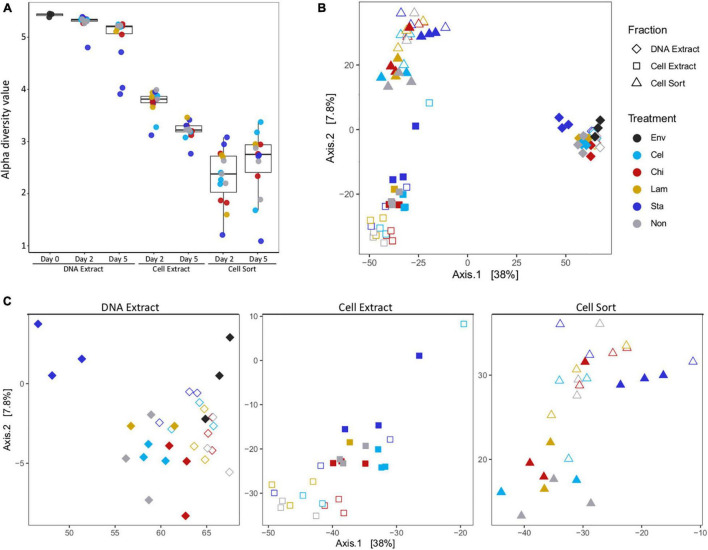
Alpha and beta diversity. Alpha diversity (Shannon index) decreased between fractions and timepoints **(A)**. Principal coordinate analysis showing differences in beta diversity between samples of all fractions **(B)** and each fraction separately **(C)**. Env: environmental sample (inoculum), Cel: cellulose, Chi: chitin, Lam: laminarin, Sta: starch, Non: no amendment. For each treatment filled symbols show day 2 and open symbols day 5.

Alpha diversity indices showed a decrease in diversity from the extractable DNA to the cell extractable fraction consistent for both timepoints analyzed (Figure 1A and Supplementary Figure 4). However, alpha diversity was lowest in the fraction recovered after cell sorting, representing only the subset of taxa that have been active in a mesocosm. Generally, the alpha diversity measure of the total community decreased with incubation time, indicating a selective enrichment of taxa within days. Interestingly, a slightly reversed trend was observed in the active community, suggesting more taxa became active over time. Community structure as displayed by principal component analysis showed overall separation by fraction (DNA extract, cell extract, cell sort) (Figure 1B). Within each fraction samples tended to cluster by timepoint. A few notably different samples included replicates amended with starch at day 5, especially in the DNA extract and cell sort fractions, one replicate amended with cellulose at day 2 in the cell extract fraction and one replicate amended with chitin at day 5 in the cell sort fraction (Figure 1C).

**FIGURE 2:**
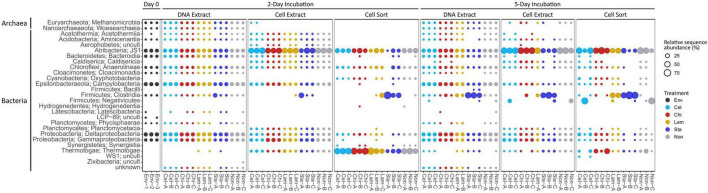
Community composition on class level showing relative sequence abundance (%) for taxa occurring with >1% in at least one sample. Env: environmental sample (inoculum), Cel: cellulose, Chi: chitin, Lam: laminarin, Sta: starch, Non: no amendment.

Based on relative sequence abundance the inoculum sediment was largely composed of Deltaproteobacteria (17%), Campylobacteria (15%), JS1 (14%), Gammaproteobacteria (8%), Bacteroidia (8%), Anaerolineae (5%), and Methanomicrobia (4%) (Figure 2). A similar, bacteria-dominated community has previously been described for Marker 14 surface sediments (Ramírez et al., 2021). The community in mesocosms recovered in the DNA extract fraction after 2 and 5 days of incubation overall showed similar composition as the inoculum sediment. Most prominent was an increase in relative proportion of Clostridia in mesocosms amended with starch (1 replicate at day 2, all replicates at day 5). Clostridia were detected initially in low abundance in the inoculum, and may have been present as endospores. In our experiments we exposed sediments to elevated temperatures after long-term cold storage, which possibly caused spore germination upon favorable temperatures followed by activity and rapid growth upon substrate availability. The microbial community might experience similar conditions in their natural environment, where, due to the high dynamics in hydrothermal activity, sediment temperatures fluctuate over time and space. For example, over a distance of a few centimeters sediment temperature often ranges from 50–100°C to 4°C (Teske et al., 2016). The cell extractable community was mostly dominated by JS1 (Atribacteria) after 2 and 5 days, except for in starch amended mesocosms where Clostridia prevailed after 5 days. The active community (cell sort fraction) at day 2 was dominated by Clostridia under starch amendment and Thermotogae under all other treatments, and generally shifted towards an Atribacteria (JS1) and Firmicutes (Clostridia and Negativicutes) dominated community at day 5. Notably, while Clostridia were prominent in incubations amended with the low complexity polysaccharides starch and laminarin, Negativicutes were prevalent in replicates without amendment or with cellulose amended.

Altogether, we observed a strong contrast between the microbial community recovered via DNA extraction and cell extraction. A lower alpha diversity index in cell extracts compared to DNA extracts indicates a preferential recovery of taxa in cell extraction procedures. Cell extractions are potentially biased and cell recovery is a frequent challenge, in particular for complex sample types such as organic carbon-rich hydrothermal sediments, which likely explains the observed differences in community structure (Morono et al., 2013; Doud and Woyke, 2017; Hatzenpichler et al., 2020). Improved cell separation techniques might lead to different observations than reported here; however, a comparison of cell extraction procedures was outside the scope of our study, which was limited by the amount of available sediment material. Further, a size selection (35 μm filtration) prior to FACS, necessary to avoid clogging of the sample line, impeded the detection of larger cell aggregates and possibly other morphologies such as long filaments in the active fraction.

Besides possible biases introduced during cell extract preparation, extracellular DNA, only represented in the DNA extract may contribute to discrepancies in community composition (Couradeau et al., 2019). Deep-sea sediments are known to contain extracellular DNA, which is also considered an important macromolecular carbon source for the heterotrophic microbial community including in Guaymas Basin sediments (Jørgensen and Jacobsen, 1996; Dell’Anno and Danovaro, 2005; Corinaldesi et al., 2007; Perez Castro et al., 2021), but the effect of extracellular DNA on microbial diversity surveys is disputed and not well established (Lever et al., 2015; Carini et al., 2016; Ramírez et al., 2018; Torti et al., 2018).

### Effect of Substrate Amendment on Abundant Populations

**FIGURE 3:**
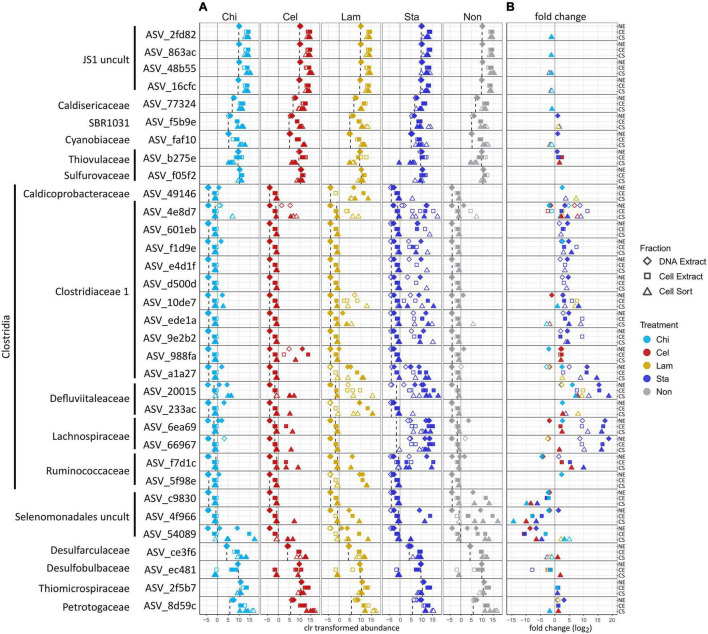
Effect of polysaccharide amendment on abundant ASVs (i.e., occurring with >3% relative sequence abundance in at least one sample). Center log ratio (clr) transformed abundance of ASVs **(A)**. Note that clr values indicate abundance of an ASV as relative to the mean abundance of all ASVs in a sample with positive clr values indicating abundance greater than the mean abundance. Dashed line: mean clr transformed abundance of an ASV in the environmental sample (inoculum, day 0). Log_2_ fold changes >1 between no amendment and each substrate treatment for each ASV **(B)**. Gray line represents a fold change of 0 (i.e., no difference between no amendment and substrate amendment). No data are available for Lam at day 5 as replicate C was lost and analysis relied on triplicate samples. Diamonds: DNA extract, squares: cell extract, triangles: cell sort, open symbols: day 2, filled symbols: day 5. Cel: cellulose, Chi: chitin, Lam: laminarin, Sta: starch, Non: no amendment.

To further characterize the community members responding to polysaccharide amendment, we analyzed the frequency of abundant ASVs (>3% relative sequence abundance in at least one sample) across fractions and treatments (Figure 3A) and tested the effect of substrate amendment by comparing the differential abundance of individual ASVs between no amendment and each substrate treatment (Figure 3B). Notably, low number of replicates and variation between replicates limited the statistical significance of our differential abundance analysis, although large fold changes were observed (Supplementary Table 2). We identified 33 abundant ASVs, which together represented a large proportion of the microbial community, encompassing a relative sequence abundance of 31% in the environmental sample and up to 97% in the active community of mesocosms, which often was dominated by only a few ASVs (Supplementary Table 2). Most of the abundant ASVs (17) affiliated with Clostridia representing six families: *Clostridiaceae* 1 (10 ASVs), *Defluviitaleaceae* (2 ASVs), *Lachnospiraceae* (2 ASVs), *Ruminococcaceae* (2 ASVs), and *Caldicoprobacteraceae* (1 ASV). All Clostridia-affiliated ASVs were initially detected in low abundance in the inoculum sediment but generally increased in proportions in the total and active community in mesocosms. Amendment with the more accessible storage polymers starch and laminarin resulted in the fasted and strongest response observed for several ASVs. In particular ASVs affiliated with the *Defluviitaleaceae* (2) and *Lachnospiraceae* (2) showed high fold changes (Figure 3B) upon starch amendment. *Defluviitaleaceae*-affiliated ASV_20015 was detected at elevated frequency in the active fraction of substrate amended incubations but not unamended controls, showing a broad response to all polysaccharides with the highest detected fold change and highest relative sequence abundance (80%) in response to starch amendment (Figure 3B and Supplementary Table 2). While many ASVs affiliated with the *Clostridiaceae* 1 occurred mostly in laminarin and starch amended incubations, ASV_4e8d7 was detected at elevated proportion in the active fraction under all treatments including unamended controls (Figure 3A); activity and growth, however, were stimulated by substrate availability (Figure 3B). This indicates that some members of the *Clostridiaceae* 1 family may utilize the substrates available in the inoculum, as Guaymas Basin sediments are naturally rich in organic and inorganic compounds (Calvert, 1966; Von Damm et al., 1985; De la Lanza-Espino and Soto, 1999). The response to chitin and cellulose amendment was generally less pronounced and limited to a few Clostridia ASVs. This suggests longer response times for these more complex structural polymers, which would be better captured with extended incubation times. ASV_988fa (*Clostridiaceae* 1) specifically responded only to chitin amendment (Figure 3B), suggesting specialized degradation capacities in members of this lineage. Besides Clostridia, other Firmicutes-affiliated ASVs belonging to an uncultured Selenomonadales clade showed strong increase in proportions, most notably in the no amendment control, and thus, no strong substrate specific response was detected.

Other abundant ASVs were affiliated to Atribacteria (JS1) (4 ASVs) which showed high initial proportions in the inoculum but little variation in mesocosms. ASVs affiliating with *Petrotogaceae* (1), SBR1031 clade (Anaerolineae) (1), *Desulfarculaceae* (1) and *Cyanobiaceae* (1) increased in the active fraction across all treatments after 2 days relative to their proportion in the inoculum. This indicates that their initial activity likely resulted from utilization of substrates supplied with the sediment and was promoted by the switch to elevated incubation temperatures but was not sustained possibly due to substrate limitation. Notably, Thermotogae were detected with the highest relative sequence abundance in the active fraction of most mesocosms after 2 days (Figure 2) but the abundant Thermotogae-affiliated ASV (ASV_8d59c) did not show a strong substrate specific response. However, Thermotogae are widespread in marine hydrothermal sediments and some members have recently been implicated in polysaccharide degradation in Guaymas Basin sediments (Perez Castro et al., 2021). In contrast, ASVs affiliated with *Thiomicrospiraceae* (1), *Thiovulaceae* (1), *Sulfurovaceae* (1) and *Desulfobulbaceae* (1), while abundant in the inoculum, decreased in proportion in the active fraction, suggesting that these organisms initially did not benefit from selected incubation conditions. However, their activity may be promoted once intermediates of polysaccharide degradation are available, possibly requiring incubation times longer than performed here. For example, production of H_2_ during polysaccharide degradation as previously observed in anoxic Guaymas Basin sediments, may favor the activity and growth of sulfate reducers (Perez Castro et al., 2021).

Overall, an increase in proportion of Clostridia-affiliated ASVs in mesocosms was observed in all fractions, and was often detected in the cell sorted fraction (day 2) prior to the DNA extractable fraction (day 5), which shows that BONCAT-FACS can reveal changes in metabolic activity before changes in cell abundance (i.e., cell division) occur. In contrast, an analysis of the DNA extractable community detects responding taxa only after DNA replication and cell division have occurred. However, both approaches produce compositional sequence data and do not provide insight into relative cell abundances.

### Potential Implications for Polysaccharide Degradation in Hydrothermal Sediments

During a relatively short incubation time, the Guaymas Basin sediment microbial community showed activity and growth when supplied with polysaccharides, indicative of the capacity to quickly (within days) synthesize the enzymatic machinery necessary to metabolize preferably low complexity storage polymers. Notably, Clostridia showed the most prominent response to substrate amendment (Figure 3B) and were frequently detected with increased proportions in all treatments but were low in abundance in the initial sediments (Figures 2, 3A) as previously observed (Perez Castro et al., 2021). In the sediments used for our experiment dormant Clostridia endospores likely endured long-term cold (4°C) storage and germinated upon exposure to elevated temperatures (33°C) prior to substrate addition. Previously, the germination of thermophilic, endospore-forming bacteria including Firmicutes and their activity in organic matter mineralization have been demonstrated when cold marine sediments were exposed to elevated temperatures (Isaksen et al., 1994; Hubert et al., 2009, 2010). Our results also suggest that while temperature may induce germination the magnitude of activity and growth likely depends on the availability of suitable substrates as a stronger response was observed in polysaccharide amended incubations compared to unamended controls. Future BONCAT-FACS experiments testing the effect of polysaccharide amendment on freshly recovered sediments (i.e., no long-term cold storage) over a range of temperatures including at 4°C would provide extended insights into the populations active in the environment under different temperature regimes.

Altogether, in sediments exposed to hydrothermal activity such as in Guaymas Basin temperature induced germination of Clostridia spores may frequently occur resulting in these organisms becoming active and important in community function as primary polymer degraders for as long as temperature regimes permit their metabolism. This suggests that a flexible lifestyle between metabolic activity and dormancy is an effective strategy for long-term survival in environments with extreme and fluctuating physicochemical conditions. Thus, in hydrothermally influenced sediments temporal and spatial fluctuations in temperature and substrate availability will constrain metabolic activity and growth resulting in dynamic microbial communities.

## Conclusion

Our study investigated the effect of polysaccharide addition on the total and active microbial communities in anoxic hydrothermal sediments. We identified a diverse, yet initially minor population of Clostridia rapidly responding with activity and growth to the availability of primarily low complexity polymers, starch and laminarin. Clostridia likely persisted in the sediments as spores during long-term cold storage and their germination in mesocosms was initiated by a switch to elevated temperatures. This indicates that in environments with fluctuating physicochemical regimes and diverse community assemblies, such as in Guaymas Basin sediments, taxa that are low in abundance or possibly dormant, including spore-forming Clostridia, may frequently assume important functions in a heterotrophic community. Consequently, time resolved correlative analysis of community composition and physicochemical parameters together with experimental investigations into metabolic activity are imperative to identify environmentally relevant taxa and assess community function.

Our study evaluated the application of BONCAT-FACS to trace metabolic responses in a hydrothermal deep-sea sediment community and highlighted the prominent challenges associated with this method when applied to a unique sample type. The outcomes will inform experimental design for future studies on the *in situ* activity and functional role of uncultured taxa in Guaymas Basin and other deep-sea sediments. In particular, cell recovery from the organic-rich sediment matrix and high diversity of the microbial community limited the comparability between DNA and cell extractable fractions and subsequently the encompassing detection of active taxa. Further, the detection of a selective effect of substrate amendment was initially limited (day 2) possibly due to the naturally high organic matter content of Guaymas Basin sediments and was more pronounced after prolonged incubation (day 5), suggesting that time of substrate exposure is critical (Calvert, 1966; Von Damm et al., 1985; De la Lanza-Espino and Soto, 1999; Zheng et al., 2021). The statistical power of our analysis was qualified by a combination of low replication and high variability between triplicates, potentially due to a preferential response of minor populations in individual replicates. Thus, cell recovery difficulties together with high cell quantity requirements and elevated replication demand challenge both applicability and throughput of BONCAT-FACS, especially when available sample material is limited. However, upon careful optimization and integration into combinatorial approaches, BONCAT-FACS expands available methods with prospect for wider implementation in microbial ecology research.

## Data Availability Statement

The datasets presented in this study can be found in online repositories. The names of the repository/repositories and accession number(s) can be found in the article/Supplementary Material.

## Author Contributions

VK and RH designed this study. VK performed experiments and data analysis. VK, RS, and NR developed fluorescence-activated cell sorting protocols. VK wrote the manuscript which was edited by all authors.

## Conflict of Interest

The authors declare that the research was conducted in the absence of any commercial or financial relationships that could be construed as a potential conflict of interest.

## Publisher’s Note

All claims expressed in this article are solely those of the authors and do not necessarily represent those of their affiliated organizations, or those of the publisher, the editors and the reviewers. Any product that may be evaluated in this article, or claim that may be made by its manufacturer, is not guaranteed or endorsed by the publisher.
