# Supplementary material for: Microbial Community Response to Polysaccharide Amendment in Anoxic Hydrothermal Sediments of the Guaymas Basin
Source: Front Microbiol. 2021 Dec 10;12:763971. doi: 10.3389/fmicb.2021.763971 (PMC8703129; doi:10.3389/fmicb.2021.763971)
Supplement: Supplementary file 1 [file Data_Sheet_1.pdf]

Supplementary Information

Microbial community response to polysaccharide amendment in anoxic hydrothermal sediments of the Guaymas Basin

Viola Krukenberg*, Nicholas J. Reichart, Rachel L. Spietz, Roland Hatzenpichler*

*Correspondence to viola.krukenberg@montana.edu and roland.hatzenpichler@montana.edu

SI Table 1. Temperature profiles recorded with *Alvin's* heat flow probe. The upper limit of thermal sensors was reached at a depth of 35 cm (>115°C). n.d.: not determined, probably close to seawater temperature (4ºC).

Sediment depth (cm)	Temperature profiles (°C)	
	T1	T2
0	n.d.	n.d.
5	12.4	15.8
10	33.3	32.8
15	53.7	50.9
20	74.1	69.3
25	92.2	88.2
30	110.4	107.6
35	>115	>115
40	>115	>115
45	>115	>115

SI Table 2. Relative sequence abundance, differential abundance and sequences of abundant ASVs. See separate data sheet.

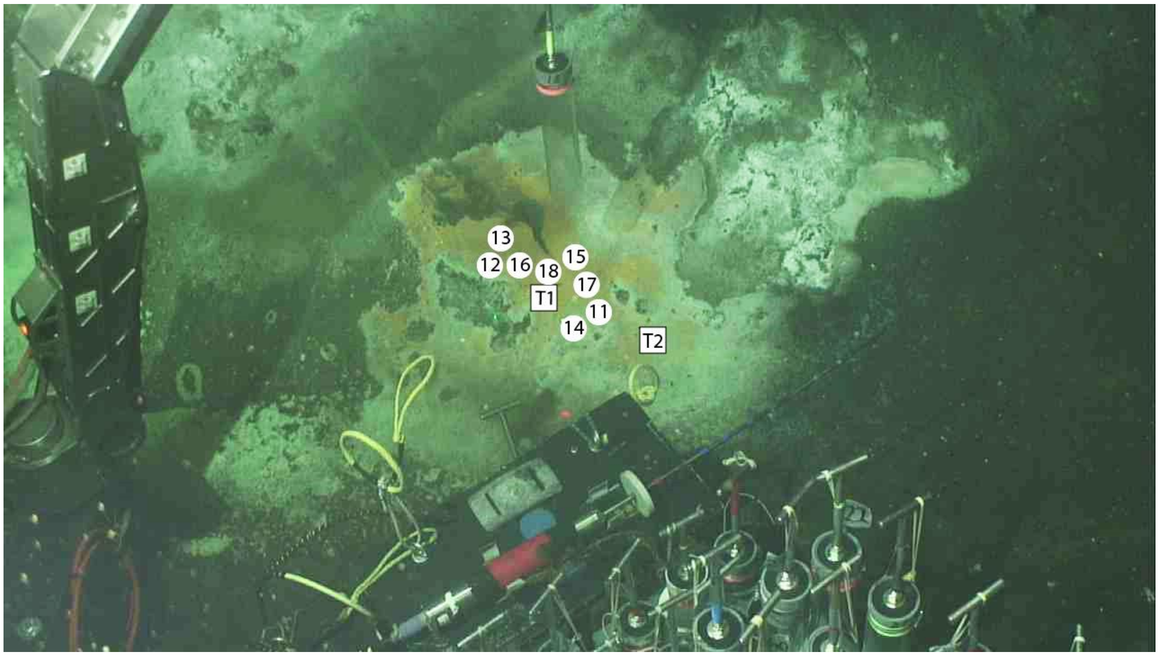

SI Figure 1. Image of the sampling site at Marker 14 in the Guaymas Basin. Eight cores (11-18) were collected from hydrothermally influenced sediment underneath *Beggiatoa* mats. Two temperature profiles (T1, T2) were recorded in the adjacent sediment (see SI Table 1). Cores 11 and 12 with corresponding temperature profile T1 were used in this study. Image courtesy of the Woods Hole Oceanographic Institution, from R/V *Atlantis* research cruise AT42-05 (November 2018) *Alvin* Dive 4998.

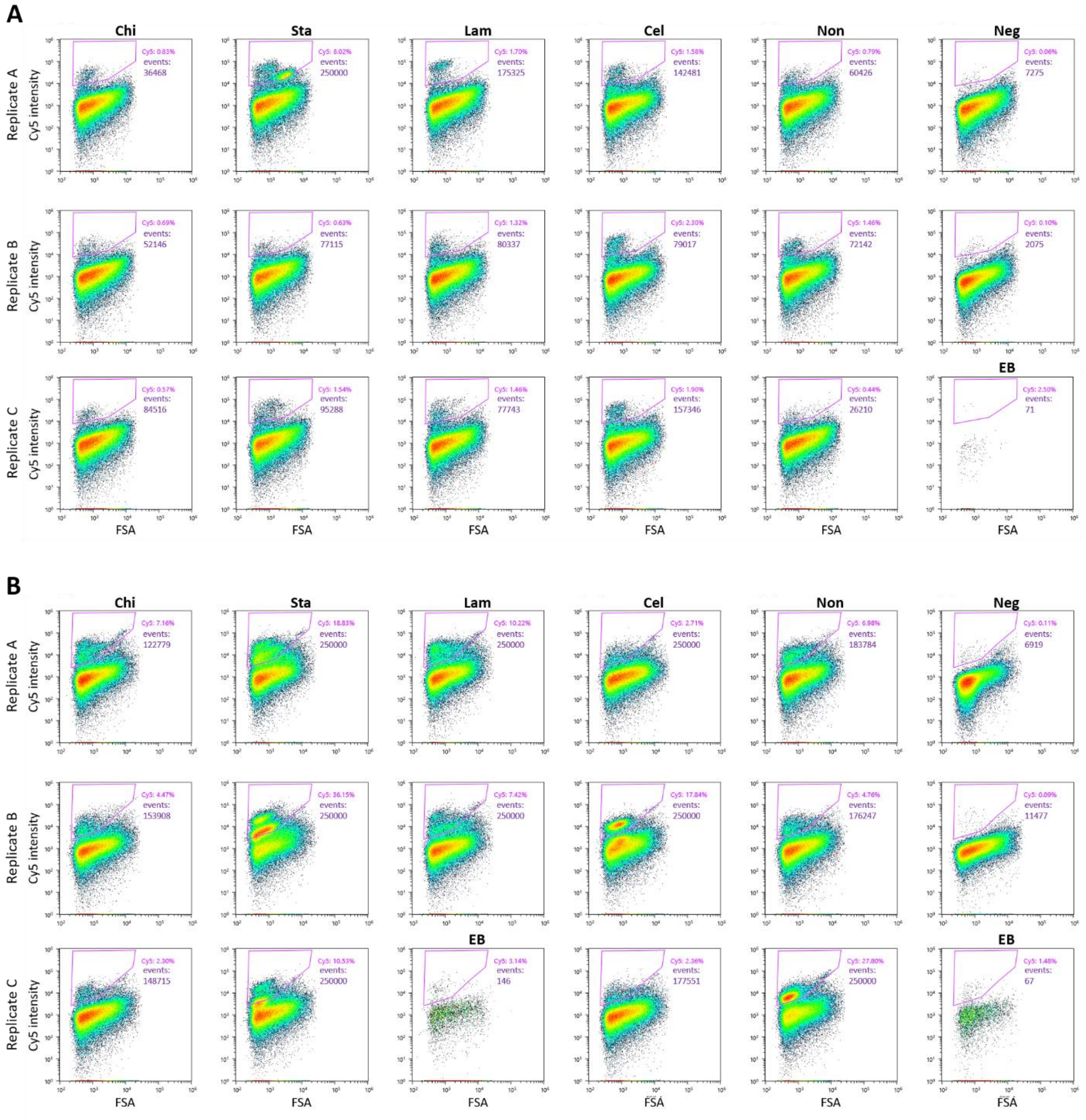

SI Figure 2. FACS plots for samples processed in this study after 2 days (A) and 5 days (B) of incubation. Samples without HPG amendment (BONCAT negative) were used to draw gates (purple polygon) to sort active (BONCAT positive) fractions. The percentage of labeled events and the total events sorted are indicated on each plot. Replicate C of Lam was lost at the second timepoint (B). Chi: chitin, Sta: starch, Lam: laminarin, Cel: cellulose, Non: no substrate amendment, Neg: no HPG and no substrate added (control for background fluorescence), EB: extraction blank using sterile 1 x PBS instead of a sample, FSA: forward scatter area.

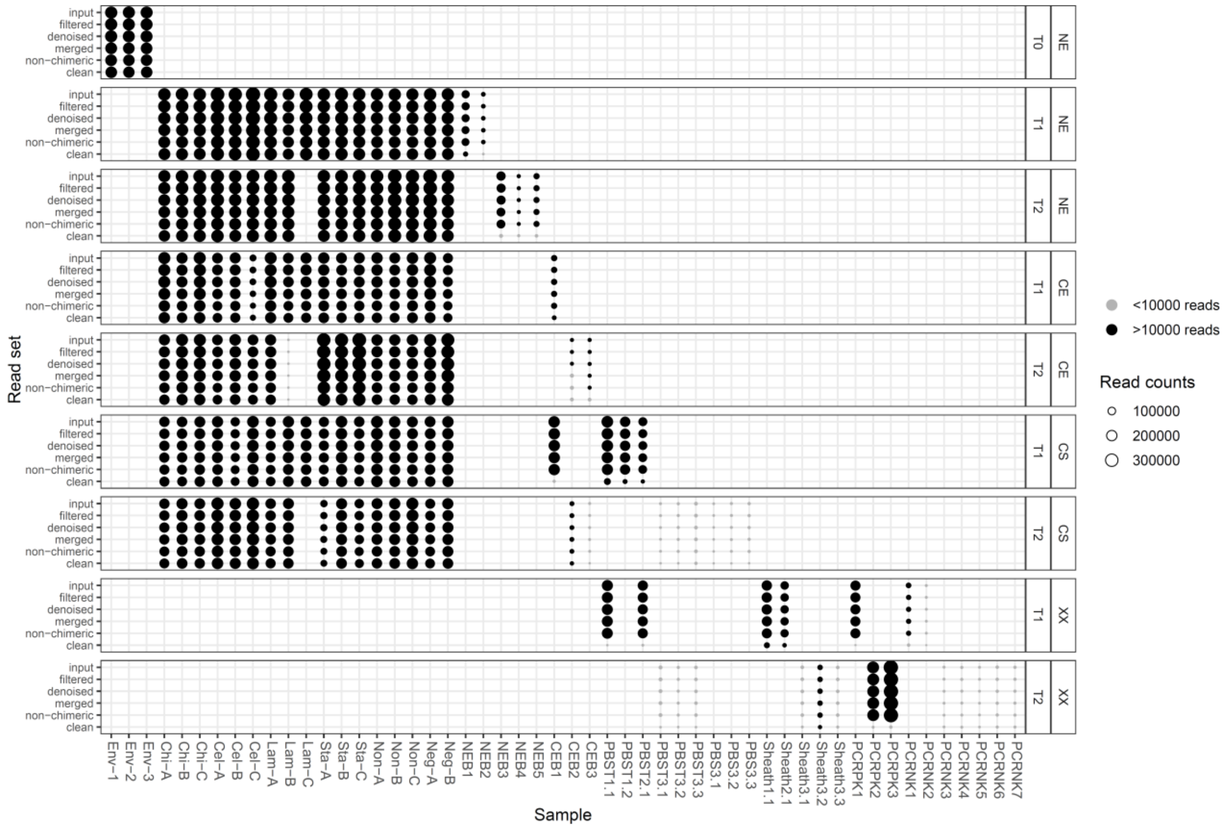

SI Figure 3. Overview of amplicon data preprocessing. Samples are grouped by fraction and timepoint or combined into controls and read counts at each processing step are indicated. NE: DNA extract, CE: cell extract, CS: cell sort, XX: other process controls, T0: day 0 (inoculum), T1: day 2, T2: day 5, clean: dataset after removal of contamination. Env: environmental sample (inoculum), Chi: chitin, Cel: cellulose, Lam: laminarin, Sta: starch, Non: no substrate amendment. The following controls were included: Neg: no HPG amendment, NEB: DNA extraction blank, CEB: cell extraction blank, PBS and sheath: sorter sheath fluid blanks, PCRNK and PCRPK: PCR negative and positive controls, respectively. Samples with less than 10000 reads and processing controls were removed from the dataset prior to further analysis.

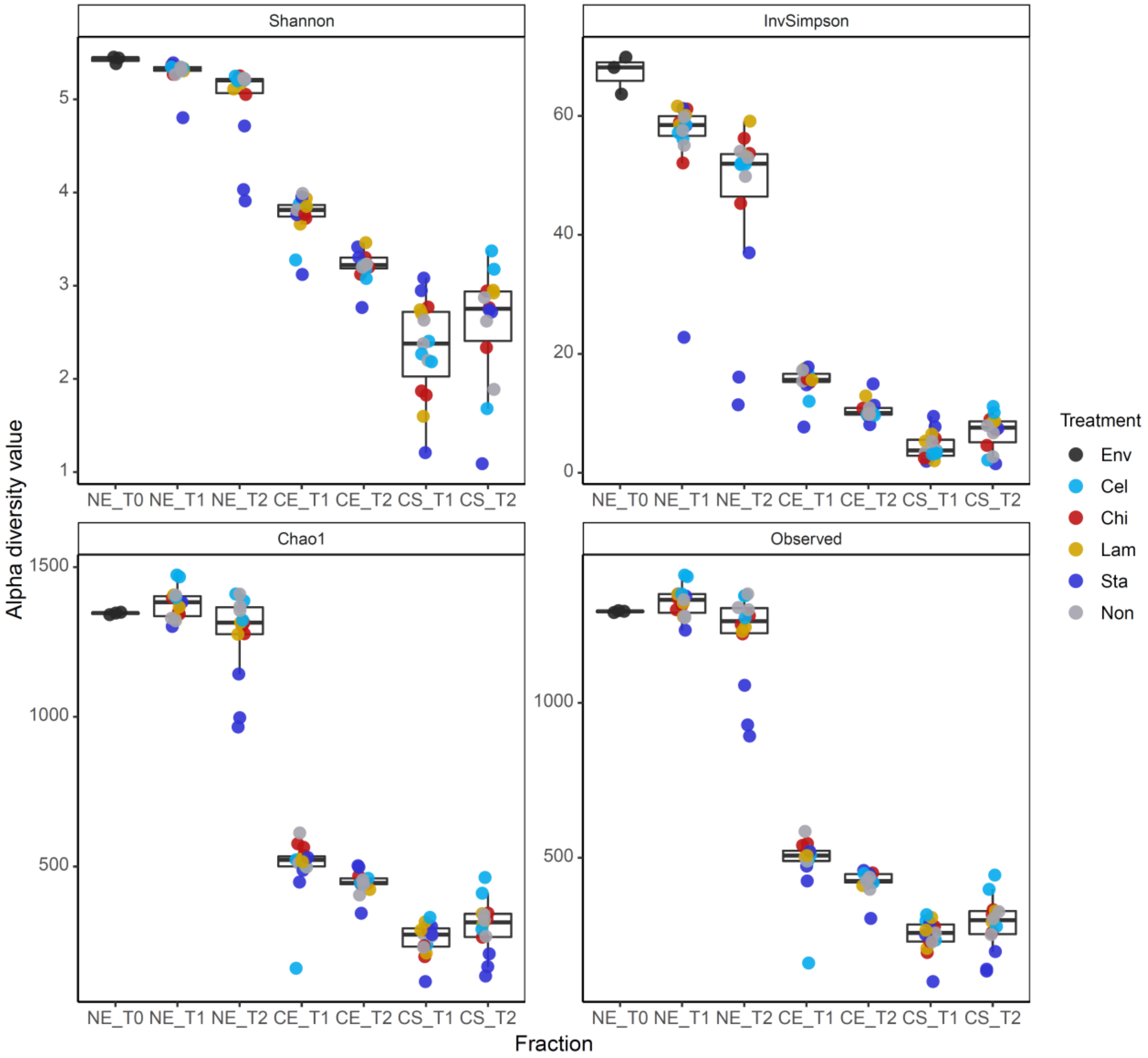

SI Figure 4. Alpha diversity measures across fractions and timepoints. Different diversity indices all indicate decreasing alpha diversity between analyzed fractions from DNA extract (NE) to cell extract (CE) to cell sort (CS). Env: environmental sample (inoculum), Cel: cellulose, Chi: chitin, Lam: laminarin, Sta: starch, Non: no amendment, T0: day 0 (inoculum), T1: day 2, T2: day 5.
